# Supplementary figures and images for: Mouse SAS-6 is required for centriole formation in embryos and integrity in embryonic stem cells
Source: eLife. 2024 Feb 26;13:e94694. doi: 10.7554/eLife.94694 (PMC10917421; doi:10.7554/eLife.94694)

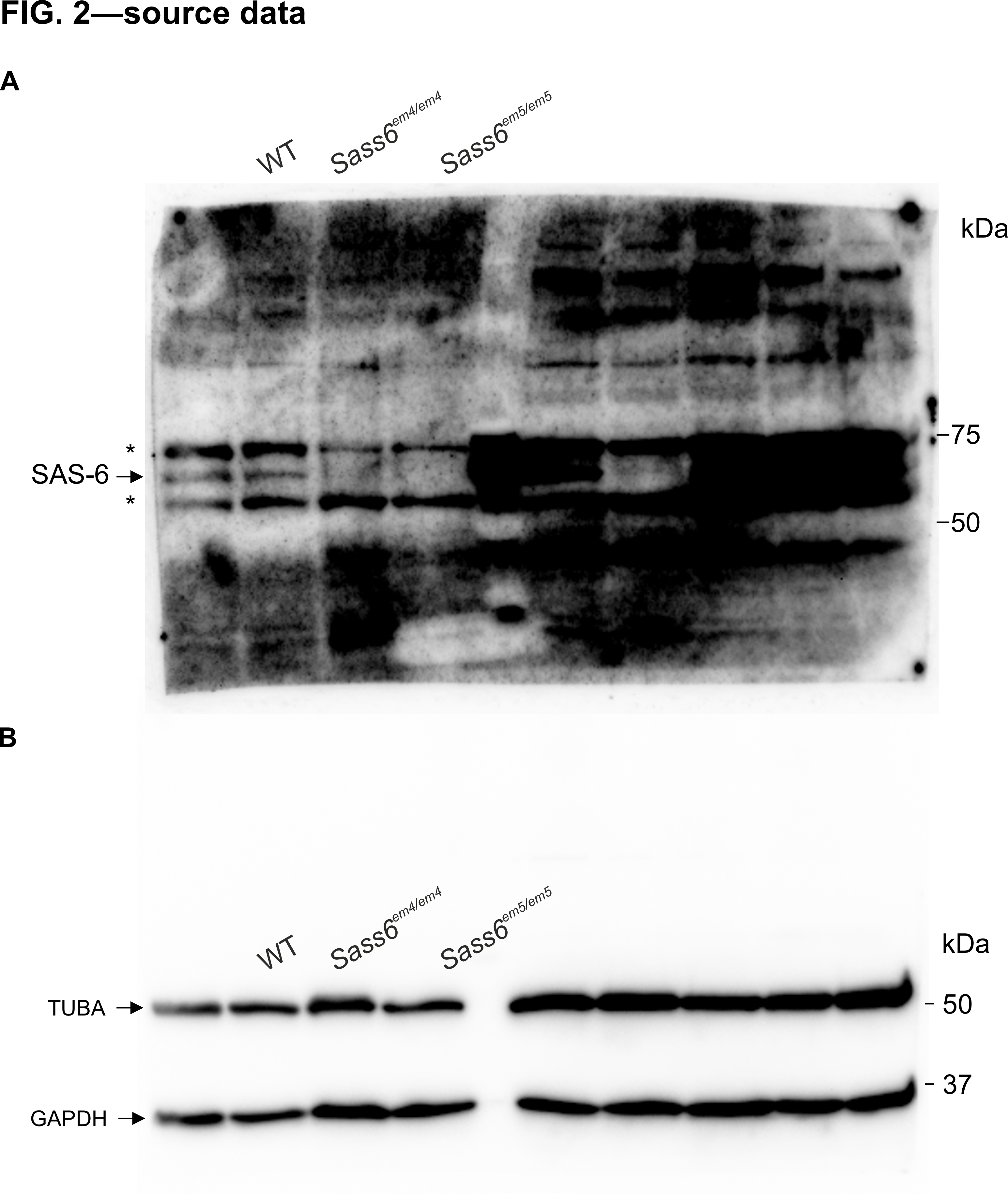

Supplement: Figure 2—source data 1. — (A) Uncropped blot from Figure 2B upper panel. Western blot analysis using a SAS-6-specific antibody on E9.5 wild-type (WT), Sass6em4/em4, and Sass6em5/em5 embryo extracts. Asterisks mark non-specific bands. (B) Uncropped blot from Figure 2B lower panel. GAPDH (and TUBA) Western blot analysis was used as a loading control. [file elife-94694-fig2-data1.zip › Figure 2-source data 1.tif]

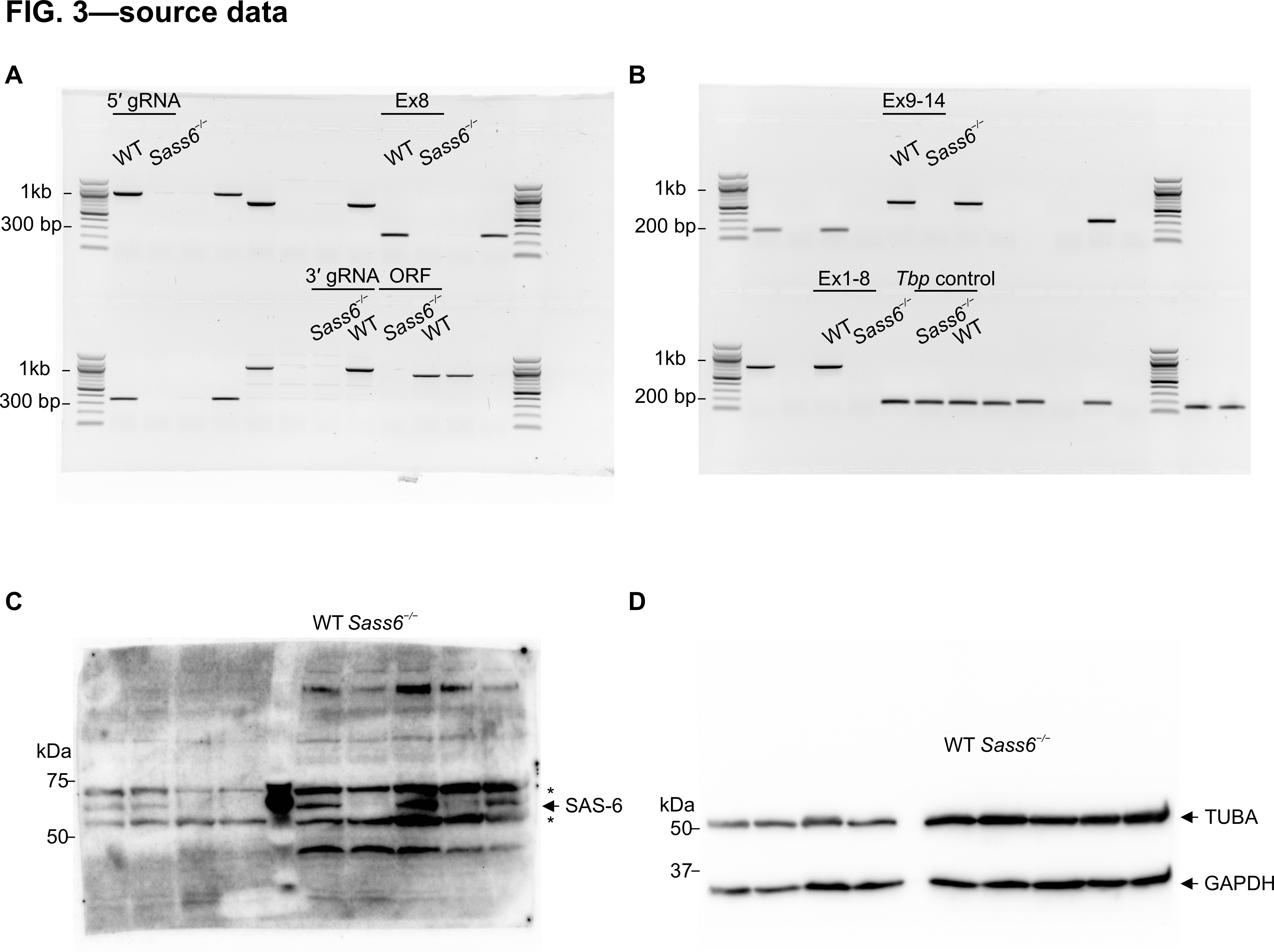

Supplement: Figure 3—source data 1. — (A) Uncropped gel picture from Figure 3A. Genomic PCR on wild-type (WT) and Sass6−/− mESCs. The picture shows the PCR products using the following primers indicated in the schematic above: 5′ gRNA (5′ F and 5′ R, band = 977 bp), Ex8 (Ex8 F and Ex8 R1, band = 281 bp), 3′ gRNA (3′ F and 3′ R, band = 992 bp), Sass6 ORF (5′ F and 3′ R, 825 bp in Sass6−/−, 34,349 bp in WT, product too long to be amplified). (B) Uncropped gel picture from Figure 3B. RT-PCR analyses of Sass6 transcripts in WT and Sass6−/− mESCs. The picture shows the PCR products from RT-PCR using the following primers: from Ex1 to Ex8 (Ex1 F and Ex8 R2, band = 734 bp), from Ex9 to Ex14 (Ex9 F and Ex14 R, band = 617 bp), Tbp Ctrl (Tbp F and Tbp R, band = 156 bp). (C) Uncropped blot from Figure 3C upper panel. Western blot analysis using a SAS-6-specific antibody on WT and Sass6−/− mESCs extracts. Asterisks mark non-specific bands. (D) Uncropped blot from Figure 3C lower panel. GAPDH (and TUBA) Western blot analysis was used as a loading control. [file elife-94694-fig3-data1.zip › Figure 3-source data 1.tif]

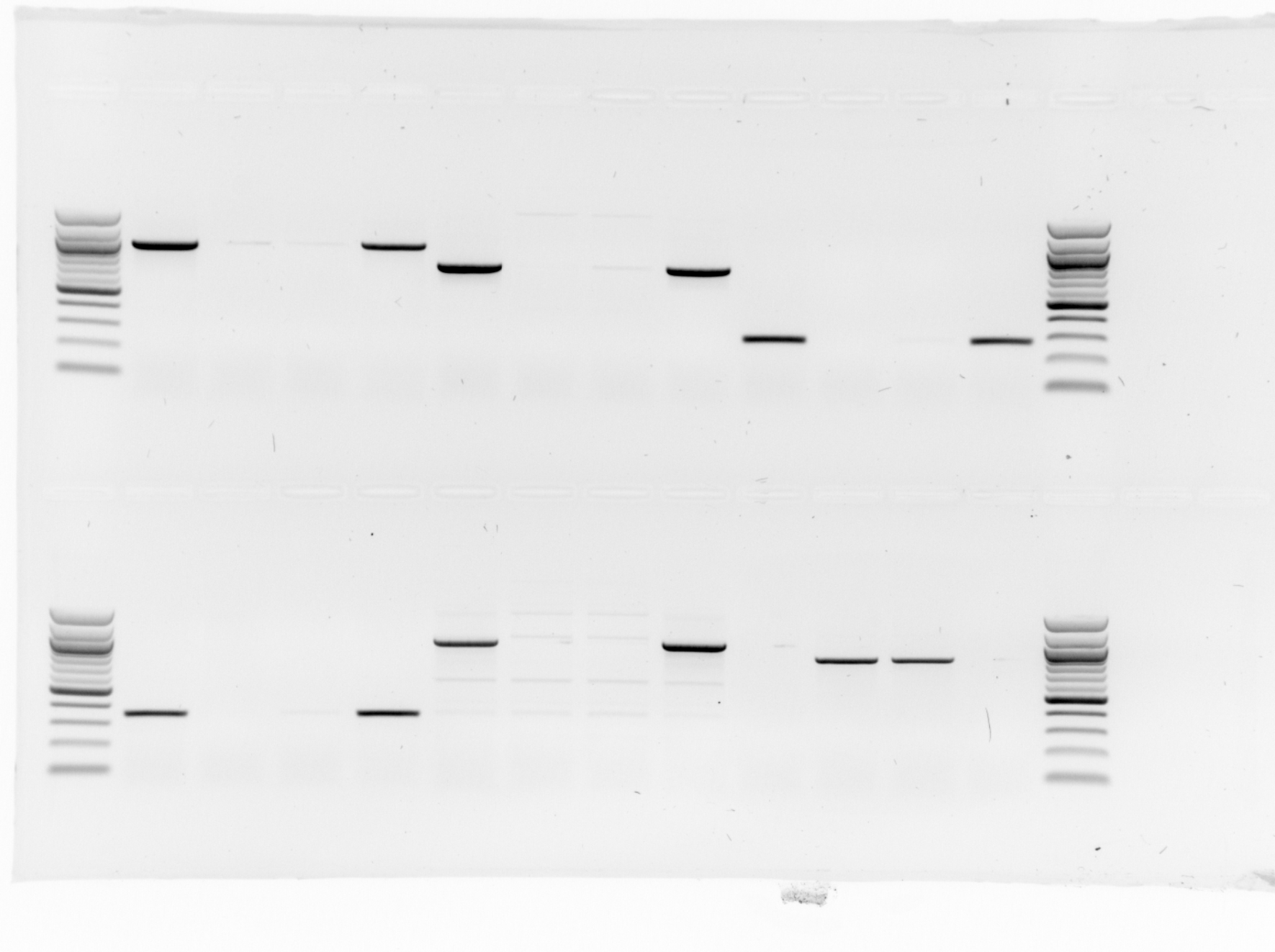

Supplement: Figure 3—source data 1. — (A) Uncropped gel picture from Figure 3A. Genomic PCR on wild-type (WT) and Sass6−/− mESCs. The picture shows the PCR products using the following primers indicated in the schematic above: 5′ gRNA (5′ F and 5′ R, band = 977 bp), Ex8 (Ex8 F and Ex8 R1, band = 281 bp), 3′ gRNA (3′ F and 3′ R, band = 992 bp), Sass6 ORF (5′ F and 3′ R, 825 bp in Sass6−/−, 34,349 bp in WT, product too long to be amplified). (B) Uncropped gel picture from Figure 3B. RT-PCR analyses of Sass6 transcripts in WT and Sass6−/− mESCs. The picture shows the PCR products from RT-PCR using the following primers: from Ex1 to Ex8 (Ex1 F and Ex8 R2, band = 734 bp), from Ex9 to Ex14 (Ex9 F and Ex14 R, band = 617 bp), Tbp Ctrl (Tbp F and Tbp R, band = 156 bp). (C) Uncropped blot from Figure 3C upper panel. Western blot analysis using a SAS-6-specific antibody on WT and Sass6−/− mESCs extracts. Asterisks mark non-specific bands. (D) Uncropped blot from Figure 3C lower panel. GAPDH (and TUBA) Western blot analysis was used as a loading control. [file elife-94694-fig3-data1.zip › Figure 3-source data 1C.tif]

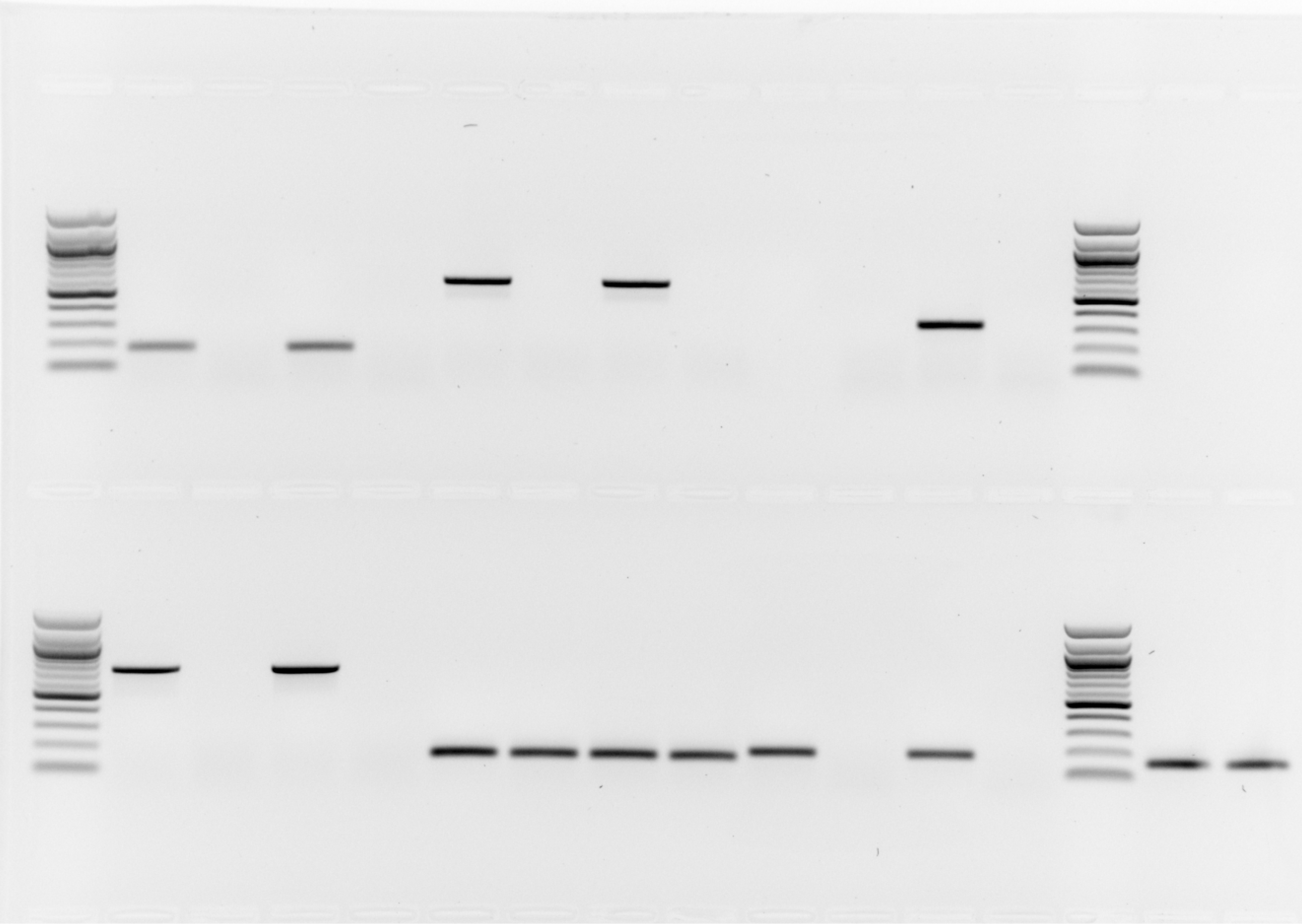

Supplement: Figure 3—source data 1. — (A) Uncropped gel picture from Figure 3A. Genomic PCR on wild-type (WT) and Sass6−/− mESCs. The picture shows the PCR products using the following primers indicated in the schematic above: 5′ gRNA (5′ F and 5′ R, band = 977 bp), Ex8 (Ex8 F and Ex8 R1, band = 281 bp), 3′ gRNA (3′ F and 3′ R, band = 992 bp), Sass6 ORF (5′ F and 3′ R, 825 bp in Sass6−/−, 34,349 bp in WT, product too long to be amplified). (B) Uncropped gel picture from Figure 3B. RT-PCR analyses of Sass6 transcripts in WT and Sass6−/− mESCs. The picture shows the PCR products from RT-PCR using the following primers: from Ex1 to Ex8 (Ex1 F and Ex8 R2, band = 734 bp), from Ex9 to Ex14 (Ex9 F and Ex14 R, band = 617 bp), Tbp Ctrl (Tbp F and Tbp R, band = 156 bp). (C) Uncropped blot from Figure 3C upper panel. Western blot analysis using a SAS-6-specific antibody on WT and Sass6−/− mESCs extracts. Asterisks mark non-specific bands. (D) Uncropped blot from Figure 3C lower panel. GAPDH (and TUBA) Western blot analysis was used as a loading control. [file elife-94694-fig3-data1.zip › Figure 3-source data 1D.tif]
