# Supplementary material for: Mouse SAS-6 is required for centriole formation in embryos and integrity in embryonic stem cells
Source: eLife. 2024 Feb 26;13:e94694. doi: 10.7554/eLife.94694 (PMC10917421; doi:10.7554/eLife.94694)
Supplement: Supplementary file 1. [file elife-94694-supp1.docx]

**Supplementary file 1.** List of used primers

| Primer | Binding site | Sequence (5`‑ 3`) |
| --- | --- | --- |
| em4 F | Intron 3 | AAGGAGCTCAGAAATTTGATGC |
| em4 R | Intron 4 | GCCAACATGAGTGCACACAA |
| em5 F | Intron 4 | CTCTTCTCCCCTTCGCTGTC |
| em5 R | Intron 5 | GCTGTTCTTGTACCACACCCT |
| 5′ F | 5′ upstream sequence | GGACTAGCGGAAGGAAGGAT |
| 5′ R | Intron 1 | CGGCTTGCCATTTCCTCATT |
| 3′ F | Exon 17 | TCGTCTTCAGCCTACTTCCC |
| 3′ R | 3′ downstream sequence | ACTCCAAATGCCCATACCCA |
| Ex8 F | Intron 7 | CTTCTGCTCTTTCTCCTTTGACA |
| Ex8 R1 | Intron 8 | TAGCCACACTAAAGCCGAGG |
| Ex1 F | Exon 1 and 2 | CAAAGACTGCGAGGAGAGGA |
| Ex8 R2 | Exon 8 | TAGCTGCCTCTAACTCGGAC |
| Ex9 F | Exon 9 | ATCCAAGAGCAAAAGGTGGC |
| Ex14R | Exon 14 | GAGATAGGGTGGCTGGTGTT |
| Tbp F | Exon 4 | CTGGAATTGTACCGCAGCTT |
| Tbp R | Exon 7 | CAGTTGTCCGTGGCTCTCTT |
